# Vaginal Microbial Colonization after Antibiotic Treatment in Women with Preterm Premature Rupture of Membranes: An Observational Cohort Study

**DOI:** 10.3390/jcm12237249

**Published:** 2023-11-23

**Authors:** Fanny Mikula, Melanie Wimmer, Alex Farr, Harald Leitich, Julia Ebner, Agnes Grill, Sonja Granser, Philipp Foessleitner

**Affiliations:** 1 Division of Obstetrics and Feto-Maternal Medicine, Department of Obstetrics and Gynecology, Medical University of Vienna, Waehringer Guertel 18-20, 1090 Vienna, Austria; fanny.mikula@meduniwien.ac.at (F.M.); alex.farr@meduniwien.ac.at (A.F.); harald.leitich@meduniwien.ac.at (H.L.); sonja.granser@meduniwien.ac.at (S.G.); 2 Department of Infection Control and Hospital Epidemiology, Medical University Vienna, 1090 Vienna, Austria; julia.ebner@meduniwien.ac.at; 3 Division of Neonatology, Pediatric Intensive Care and Neuropediatrics, Department of Pediatrics and Adolescent Medicine, Comprehensive Center for Pediatrics, Medical University Vienna, 1090 Vienna, Austria; agnes.grill@meduniwien.ac.at

**Keywords:** vaginal microbiome, preterm premature rupture of membranes, preterm birth, chorioamnionitis, early-onset neonatal sepsis, antibiotic resistance

## Abstract

Preterm premature rupture of membranes (pPROM) stands as a primary contributor to preterm deliveries worldwide, closely linked to consequential infectious peripartum complications, including chorioamnionitis and early-onset neonatal sepsis. As a prophylactic measure, individuals following pPROM routinely undergo antibiotic treatment. The aim of this study was to evaluate changes in the vaginal microbial colonization after antibiotic treatment following pPROM. Therefore, we retrospectively assessed the impact of antibiotic treatment on the maternal vaginal microbial colonization in 438 post-pPROM patients delivering before 29 gestational weeks. Vaginal samples were collected for microbiological analysis before and after antibiotic administration and analysed for seventeen pre-defined microbial groups. We observed eradication in eleven microbial groups, including beta-hemolytic streptococci group B and *Gardnerella vaginalis*. No significant reduction was found for the remaining groups, including *Escherichia (E.) coli*. Moreover, we found a notable increase in resistant bacteria after antibiotic treatment. In conclusion, broad-spectrum antimicrobial treatment exhibited substantial efficacy in eradicating the majority of pathogens in our cohort. However, certain pathogens, notably *E. coli*, showed resilience. Given *E. coli*’s prominent role in infectious peripartum complications, our findings underline the challenges in antibiotic management post-pPROM and the need to establish international guidelines, particularly regarding emerging concerns about antibiotic resistances.

## 1. Introduction

Preterm birth is defined as delivery before 37 gestational weeks. Extremely preterm infants are born before 28 gestational weeks, very preterm infants between 28 and 32 weeks, and moderate to late preterm infants are born between 32 and 37 gestational weeks [1]. Preterm birth stands as the leading cause for mortality in children under the age of five. In the year 2020, approximately 13.4 million babies were born at premature age worldwide [1].

The causes for preterm birth are diverse; it occurs either due to spontaneous labor with intact amniotic membrane, the preterm premature rupture of membranes (pPROM) or iatrogenic, early labor induction, or cesarean section due to a maternal or fetal indication [2]. The pathophysiology of spontaneous preterm birth is manifold and multiple causative factors have been identified, such as infection, inflammation, progesterone deficiency, and uterine distension [2].

In industrialized countries, the survival rates of preterm infants born at 24 gestational weeks are reported to range from 50% to 90% at 28 weeks [3]. The risk for severe complications decreases with increasing weight and gestational age at delivery [4].

PPROM is defined as the rupture of the membranes before the onset of labor and before 37 gestational weeks. This condition complicates about 3% of all pregnancies and causes approximately 25 to 30% of all preterm deliveries [2,5]. The etiology of pPROM is not yet sufficiently resolved; however, multiple risk factors contributing to pPROM have been identified, for example a pPROM in previous pregnancies, vaginal infections, invasive prenatal diagnostics, smoking, or cervical insufficiency are known to increase the pPROM risk [5,6,7]. The management of pPROM depends on several factors, including gestational age and whether or not the patient shows clinical or laboratory signs of infection [5,8]. Today’s main challenge in clinical management remains weighing the benefits of prolonging the pregnancy against the risk of amniotic infection and the associated risks for the mother and the unborn child [7]. Dependent on the gestational age, corticosteroids for lung maturation and magnesium sulphate for fetal neuroprotection are recommended prior to delivery [6,9]. A Cochrane review including 22 studies concluded that antibiotic treatment is associated with a prolongation of pregnancy and reduced maternal, as well as neonatal, morbidity, including the occurrence of infection and need for respiratory aid, even though there was no significant difference in perinatal mortality. However, due to the variability of the data, there are no international recommendations available on the use of a specific antibiotic regimen [10].

Numerous studies have investigated the vaginal microbial colonization among individuals with pPROM in recent years [11,12,13,14], yielding diverse findings. Paramel Jayaprakash et al. [12] observed a highly heterogeneous and unstable vaginal microbiome in pPROM patients. Saghafi et al. [14] investigated the endocervical microbiome within this patient cohort, identifying the most prevalent microorganisms as *Eschericia (E.) coli*, coagulase-negative staphylococci, enterococci, and *Candida* species.

In the last decades, antibiotic resistance has become an increasing challenge throughout the world, with previous antibiotic consumption being a common promoting factor [15]. According to the current literature, this issue is also strongly relevant in pPROM patients. In 2019, Li et al. [16] reported a rate of approximately 70% of ampicillin-resistant *E. coli* isolates in this collective.

This study aimed to assess the impact of routinely administered antibiotic treatment on the vaginal microbiota of patients post-pPROM and to evaluate whether antibiotic prophylaxis promotes the colonization of (multi-)resistant pathogens.

## 2. Methods

### 2.1. Study Design and Cohort

This is a retrospective cohort study, conducted at our tertiary perinatal center at the Medical University of Vienna. We included all women with delivery post-pPROM before 29 gestational weeks following antibiotic treatment who were admitted between 1 June 2009 and 31 December 2018 at our department. Those with triplet or higher-grade multiple pregnancies, as well as those with fetal chromosomal anomalies, fetal malformations, and/or congenital metabolic disorders were excluded from the analyses. This study was conducted in accordance with the Declaration of Helsinki and Good Scientific Practice guidelines and approved by the ethical committee of the Medical University of Vienna (application number: 2224/2020).

PPROM was diagnosed either by visualization of amniotic fluid in speculum examination or by detection of either insulin-like growth factor binding protein 1 or placental alpha microglobulin-1 in the vaginal fluid. Vaginal swabs for microbiological analysis were then collected from the posterior fornix vaginae and the cervical canal for the detection of microbes with an according antibiogram. All swabs used were liquid multipurpose flocked swabs (eSwabs^TM^; Copan Italia S.p.A., Brescia, Italy). Culture results were interpreted as positive when they showed evidence of any microbial colonization. Additionally, a vaginal swab was assessed by Gram-staining for rapid diagnosis of vulvovaginal candidosis or bacterial vaginosis.

All women with pPROM received antibiotic treatment with 3 × 4 g of intravenous ampicillin for 6 days or 3 × 2 g intravenous cefazoline in case of an allergy to penicillin. In patients with a body weight over 90 kg, the dosage was increased to 3 × 6 g ampicillin or 3 × 3 g cefazoline. Beginning in October 2017, the antibiotic regimen was expanded by a single dose of 1 g azithromycin either intravenously or orally to cover a broader spectrum of microbes including further Gram-negative bacteria such as *Chlamydia trachomatis*. Antibiotic therapy was adapted after receiving the culture results and the respective antibiogram whenever necessary.

### 2.2. Data Collection

We collected a vaginal swab as the baseline microbial sample at the diagnosis of pPROM. Depending on the baseline culture result, follow-up swabs for microbial analysis were sampled during pregnancy. As part of our clinical routine, samples for microbial analysis were systematically collected from the placenta and amnion upon delivery, and these specimens were designated as the follow-up samples for our study. In cases where the aforementioned sample was unavailable, we used the last vaginal swab taken prior to delivery for analysis. Subsequently, a comparative analysis of bacterial and fungal colonization alterations following antimicrobial therapy was conducted between the baseline and follow-up samples. For the analysis, we collected demographic and perinatal data from obstetric databases and patient charts using the PIA Fetal Database, version 5.6.28.56 (General Electric Company, GE Viewpoint, Munich, Germany). These parameters included maternal age, singleton or twin pregnancy, gestational age at delivery, latency period between pPROM and delivery, antibiotic regimen, and application of fetal lung maturation and/or tocolysis. According to the most frequent pathogens, we pre-defined 17 microbial groups for analysis in our study; these groups are presented in Table 1.

### 2.3. Statistical Analysis

We used absolute and relative frequencies of each microbial group before and after antibiotic treatment, as well as a 95% confidence interval calculated for the relative frequencies. Changes in the vaginal microbial colonization after antibiotic treatment were evaluated by comparing each participant’s first and last culture result. The significance level was defined at *p* < 0.05, using the McNemar test for related samples. Resistant and multi-resistant pathogens were separately evaluated. We performed descriptive statistics for maternal and perinatal characteristics, with metric variables being described as mean and standard deviation (SD), and categorical variables as absolute and relative frequencies. Power calculations were not required due to the exploratory design of this study. Statistical analysis was conducted using IBM SPSS Statistics for Windows, Version 27.0 (IBM Corp., Armonk, NY, USA).

## 3. Results

### 3.1. Patient Collective

We identified a total of 471 eligible women with pPROM before 29 gestational weeks. Out of these, 33 cases were excluded due to the absence of any antimicrobial treatment. In these cases, antibiotic treatment was not administered due to various factors, with the most prevalent being rapid delivery after pPROM. The remaining 438 cases were forwarded to the analyses of our study. Out of this cohort, 79 of 438 women (18%) had a twin pregnancy, whereof 6 twin infants were excluded as at least one of the twins fulfilled the exclusion criteria. In these cases, we only included the maternal as well as the other twins’ neonatal data in the analysis.

In our study cohort, the median maternal age at delivery was 32.1 (SD ± 6) years. The 511 included neonates had a median birthweight of 929.3 (SD ± 252.7) grams. Among these, 216 (42.3%) were female, while 295 (57.7%) were male. The median latency period between pPROM and delivery was three days. Tocolysis was administered in 422 (96.3%) cases, and antenatal steroids for fetal lung maturation in 433 (98.9%) cases. Cesarean section was performed in 383 (87.4%) cases. During their admission to the neonatal intensive care unit, a total of 57 (11.2%) neonates unfortunately died. Detailed maternal and neonatal characteristics are shown in Table 2 and Table 3.

### 3.2. Microbial Swabs and Antibiotic Treatment

Baseline vaginal swabs were collected for microbial analysis from 245 of 438 women (55.9%) prior to the initiation of antibiotic treatment, yielding positive results in 240 (98%) of these cases. The follow-up swab was obtained from 396 patients (90.4%), with positive cultures observed in 247 (62.4%) women.

Among the 438 patients comprising the study cohort, 300 (68.5%) received antibiotic monotherapy. Ampicillin emerged as the most frequently used antibiotic agent, administered in 349 cases (79.7%), followed by clindamycin and azithromycin, which were administered in 86 (19.6%) and 42 cases (9.6%), respectively. A comprehensive list of all prescribed antibiotics is presented in Table 4.

### 3.3. Changes in the Vaginal Microbial Colonization after Antibiotic Treatment

We obtained comprehensive culture results both pre- and post-antibiotic therapy for 226 of 438 (51.5%) patients. In 11 out of the 17 defined microbial groups, a statistically significant reduction in the number of patients with positive culture results for the respective microbial group was observed following antibiotic treatment. No statistically significant increase was detected for any of the investigated microbial groups. The exact results are shown in Table 5.

Comparing the baseline with the follow-up swabs, we found a statistically significant reduction in the abundance of *Lactobacillus* species, coagulase-negative streptococci, *Corynebacterium* species, *Enterococcus* species, beta-hemolytic streptococci group B, and *Gardnerella vaginalis* following antibiotic treatment with respective *p*-values of <0.001. The abundance of other beta-hemolytic streptococci (*p* = 0.021) and *Viridans* streptococci (*p* = 0.007) also significantly decreased after the administration of antibiotic therapy.

No statistically significant changes were identified for *Ureaplasma* species (*p* = 0.289), *Mycoplasma hominis* (*p* = 0.549), *E. coli* (*p* = 0.627), other *enterobacterales* (*p* = 0.134), *Staphylococcus aureus* (*p* = 0.219), and Gram-positive anaerobes (*p* = 0.169).

Additionally, we analyzed cultures regarding *Candida albicans* and non-albicans *Candida*. Following antibiotic treatment and, when indicated, antifungal therapy, a statistically significant decrease in positive culture results was observed, with *p*-values of <0.001 for *Candida albicans* and 0.004 for non-albicans *Candida*. Only one patient (0.4%) exhibited a persistent colonization with *Candida* spp. during the course of treatment.

Additionally, we investigated the incidence of resistant or multi-resistant pathogens. Positive results were identified in the baseline swab and subsequently turned negative in 12 patients (5.3%), whereas in 27 patients (12%), culture results were initially negative, later transitioning to positive in the follow-up swab. This increase in resistant pathogens following antibiotic treatment achieved statistical significance with a *p*-value of 0.024. Notably, only a singular instance of a positive culture result for a multi-resistant pathogen was recorded throughout the study, occurring post-antibiotic treatment (Table 6).

## 4. Discussion

There are multiple pillars in the management of pPROM, one of which entails the administration of antibiotics to mitigate the risk of amniotic infection [5]. Our study found a significant eradication following antibiotic treatment among 11 of the 17 defined microbial groups in women with pPROM, but also the persistence of *E. coli* despite antibiotic treatment.

Among our study collective, the predominant antibiotic agent was ampicillin, followed by clindamycin and azithromycin. To date, there are no internationally recognized guidelines available defining a first-line antibiotic regimen for women with pPROM [10]. However, the existing literature suggests that a combined treatment with penicillin and macrolides might be beneficial in these cases [6]. Moreover, it is suggested that the combination of amoxicillin and clavulanate should be avoided due to its increased risk for neonatal necrotizing enterocolitis [17]. At our center, we expanded the antibiotic regimen to include clindamycin in cases with severe vaginal dysbiosis or bacterial vaginosis following international recommendations [18].

The effect of the antibiotic treatment on the vaginal ecosystem in pregnancy has been a topic of scientific interest for many years. In a study conducted by Stokholm et al. [19], the authors analyzed the vaginal microbiome in pregnant women undergoing antibiotic treatment for different indications. They found a significant increase in the colonization with *Staphylococcus* spp., particularly among those treated for urinary tract infections [19]. Consonantly, Norinder et al. [20] reached similar conclusions; their analysis also identified an increased vaginal colonization with *E. coli* subsequent to antibiotic treatment for respiratory tract infections [19]. To date, there are only few studies examining the impact of antibiotic treatment on the vaginal microbial colonization in patients experiencing pPROM. Bennet et al. [21] conducted a comprehensive review of multiple studies assessing the efficacy of erythromycin in pPROM patients. Their synthesis indicated that erythromycin is insufficient in eradicating pathogens and reinstating a balanced vaginal microbiome within this patient cohort. In a study by Baldwin et al. [22], no significant eradication of *Lactobacillus* spp. or *Prevotella* spp. was observed following antibiotic treatment with ampicillin, amoxicillin, or azithromycin. The study did, however, reveal a significant reduction in certain species such as *Weeksella* or *Lachnospira* spp., alongside an increase in others, including *Peptostreptococcus* and *Tissierellaceae ph2*.

Our assessment of post-pPROM women revealed a substantial eradication of various microbial groups, including *Gardnerella vaginalis* and beta-hemolytic streptococci Group B. These findings indicate a normalization of a disrupted vaginal microbial colonization and the elimination of vaginal infections. However, we also found a significant reduction in the colonization with *Lactobacillus* spp., indicating an iatrogenic disturbance in cases with a normal vaginal microbiota, potentially increasing susceptibility to pathogen overgrowth [23]. It is noteworthy to mention that, despite these findings, we found no increase in the positive culture results for any of the microbial groups that we observed. The literature suggests an increased risk, particularly for fungal colonization and infection following antibiotic exposure [24,25]. However, contrary to the existing body of literature, our data revealed no increase in the vaginal colonization with *Candida* spp. in post-pPROM women undergoing antibiotic prophylaxis. Additionally, in cases of pre-existing fungal colonization, our results show that, when coupled with an appropriate antifungal treatment, a significant eradication of *Candida* spp. can still be achieved.

Chorioamnionitis is a dreaded complication in women following pPROM, which is thought to derive from ascending vaginal pathogens after the amniotic barrier is diminished [26]. Frequently associated pathogens encompass, among others, Group B streptococci, *Mycoplasma* spp., *Gardnerella vaginalis*, *E. coli*, and *Candida* spp. [27]. The primary objective of antibiotic prophylaxis in patients experiencing pPROM is the prevention of chorioamnionitis. This measure not only prolongs pregnancy duration, but also enhances both maternal and neonatal health [17]. While the administered broad-spectrum antibiotic agents that were used in our study collective, with ampicillin being the most frequently used substance, significantly eradicated pathogens that are associated with chorioamnionitis, certain relevant microorganisms, such as *E. coli* or *Mycoplasma hominis*, were not sufficiently eliminated. This observation aligns with the existing literature: In 2019, Li et al. [16] reported a rate of approximately 70% of ampicillin-resistant *E. coli* isolates in pPROM patients.

As a result of pPROM and chorioamnionitis, affected infants are at risk to acquire early onset neonatal sepsis (EONS), diagnosed in about 20 out of 1000 infants born prior to 29 weeks of gestation [28]. In our cohort, 13 out of 501 (2.6%) neonates showed positive blood culture results postpartum. The causative pathogens for this disease predominantly derive from the maternal genital tract, with *Escherichia coli* and group B streptococci being the most frequent [29]. Pathogens such as *Hemophilus influenzae*, coagulase-negative staphylococci, *Candida* spp., *enterobacteria*, *listeria*, or anaerobes are also associated with EONS but are known to be less prevalent [29]. EONS is a critical condition with a high mortality rate among affected newborns, and it is significantly increased in premature infants. Thereby, the mortality rate for infants with a birthweight below 1500 g is approximately 35% [28]. Our findings demonstrate significant efficacy in eradicating numerous relevant pathogens, including beta-hemolytic streptococci group B, whereas there was no significant eradication of *E. coli* by the prescribed antibiotic regimen in our patient cohort. These findings are especially relevant with regards to the related literature: Tsai et al. [30] conducted a study revealing that 79% of all *E. coli* isolates in newborns with *E. coli*-associated EONS exhibited ampicillin resistance.

Moreover, there are studies suggesting that colonization with antimicrobial-resistant pathogens may increase in women with pPROM after antibiotic treatment [31]. Similarly, available data indicate an elevated risk of EONS associated with the presence of resistant pathogens subsequent to maternal antibiotic treatment during pregnancy, though data in this area are notably inconsistent [32,33,34]. To address this question, we additionally analyzed our data for resistant and multi-resistant pathogens, and we found a significant increase in antibiotic-resistant microorganisms following antibiotic therapy. This is a crucial finding that stands in accordance with the currently available literature, indicating a consistent increase in the prevalence of both resistant and multi-resistant pathogens, which are known to be responsible for complications such as EONS and chorioamnionitis [35,36,37]. The increase in microbial resistance is an alarming reality, which might pose a significant threat due to missing therapy alternatives and thereby increasing mortality rates [38].

As previously mentioned, presently, there is no international consensus regarding the optimal first-line antibiotic treatment for patients post-pPROM [10]. However, given the increasing rates of microbial resistance as well as [38] *E. coli*-associated EONS [39], these findings warrant careful consideration. Wolf et al. [37] thus proposed the periodic conduct of antibiotic sensitivity profiles to regularly update national guidelines for the antibiotic treatment of patients following pPROM.

We are aware of the strengths and limitations of our study. Firstly, this is a retrospective study and, unfortunately, the complete microbial sets were only available for 226 of our cases. While this cohort still provided a substantial sample size for facilitating robust statistical significance for our analysis, it is imperative to recognize the potential for performance and selection bias due to the retrospective study design. As a strength, the administration of a diverse range of antibiotic agents in our cohort enhances the generalizability of our findings beyond local treatment guidelines. The broad spectrum of the microbial groups that we analyzed also allows a comprehensive presentation of the changes in the vaginal ecosystem after antibiotic treatment. Regrettably, due to insufficient data, our analysis did not extend to multi-resistant pathogens, and we are aware that this is a clear limitation of our work.

## 5. Conclusions

Antibiotic treatment in women with pPROM eradicates the majority of microbial groups with a notable persistence of *E. coli*, which emphasizes the challenge of achieving comprehensive microbial eradication in this challenging situation. However, in the light of the “antibiotic stewardship”, we consider antimicrobial resistance as a harmful downside of unnecessary or incorrect antibiotic treatments that have the potential to negatively affect both the mother and the newborn. Internationally validated guidelines are warranted to clearly define a regimen of the respective first-line antibiotic agents in women with pPROM. Our study herewith highlights the necessity to establish these, particularly in the context of emerging concerns about antibiotic resistances.

## Figures and Tables

**Table 1:** Predefined microbial groups for the analysis of changes in the vaginal microbial colonization following antibiotic treatment post-pPROM.

Microbial Groups
*Lactobacillus* species
Coagulase-negative staphylococci
*Ureaplasma* species
*Mycoplasma hominis*
*Corynebacterium* species
*Enterococcus* species
Beta-hemolytic streptococci group B
Other beta-hemolytic streptococci
*Viridans* streptococci
*Escherichia coli*
Other *enterobacterales*
*Gardnerella vaginalis*
*Candida albicans*
Non-albicans *Candida*
*Staphylococcus aureus*
Gram-positive anaerobes
Gram-negative anaerobes

**Table 2:** Maternal characteristics, treatment details, and culture results of the 438 included women with pPROM within our study cohort.

Maternal Characteristics	Patient Collective (*n* = 438)
Maternal age in years, mean (SD)	32.1 (±6)
Gestational age at delivery in completed weeks, *n* (%)	
23	39 (8.9%)
24	61 (13.9%)
25	74 (16.9%)
26	74 (19.9%)
27	81 (18.5%)
28	52 (11.9%)
29	57 (13.0%)
Latency period between pPROM and delivery in days, median (q25; q75)	3 (1; 12)
Duration of antibiotic therapy in days, median (q25; q75)	6 (4; 8.25)
Delivery by cesarean section, *n* (%)	383 (87.4%)
Vaginal delivery, *n* (%)	54 (12.6%)
Twin delivery, *n* (%)	79 (18.0%)
No. of patients who received tocolysis, *n* (%)	422 (96.3%)
No. of patients who received fetal lung maturation, *n* (%)	433 (98.9%)
Baseline culture available, *n* (%)	245 (55.9%)
Baseline culture positive, *n* (%) (*n* = 245)	240 (98.0%)
Baseline culture negative, *n* (%) (*n* = 245)	5 (2.0%)
Follow-up culture available, *n* (%)	396 (90.4%)
Follow-up culture positive, *n* (%) (*n* = 396)	247 (62.4%)
Follow-up culture negative, *n* (%) (*n* = 396)	149 (37.6%)

*n* = number of cases, SD = standard deviation, q25 = first quartile, q75 = third quartile.

**Table 3:** Neonatal characteristics including blood culture results of the 511 infants born to the 438 women with pPROM within our study cohort.

Neonatal Characteristics	Neonatal Collective (*n* = 511)
Birthweight (g), mean (SD)	929.3 (±252.7)
Sex, *n* (%)	
Female	216 (42.3%)
Male	295 (57.7%)
Neonatal blood culture available, *n* (%)	501 (98.0%)
Blood culture positive, *n* (%) (*n* = 501)	13 (2.6%)
Blood culture negative, *n* (%) (*n* = 501)	488 (97.4%)
Neonatal demise during admission at neonatal ICU, *n* (%)	57 (11.2%)

*n* = number of cases, g = grams, SD = standard deviation, ICU = intensive care unit.

**Table 4:** Administered antibiotics in the 438 women with pPROM within our study cohort throughout the course of admission in our tertiary referral-center.

Administered Antibiotics	*n* (%)
Ampicillin	349 (79.7%)
Clindamycin	86 (19.6%)
Azithromycin	42 (9.6%)
Cefazolin	29 (6.6%)
Cefuroxime	25 (5.7%)
Ampicillin/Sulbactam	22 (5.0%)
Amoxicillin/Clavulanic acid	19 (4.3%)
Clarithromycin	17 (3.9%)
Metronidazole	9 (2.1%)
Piperacillin/Tazobactam	7 (1.6%)
Josamycin	5 (1.1%)
Meropenem	3 (0.7%)
Penicillin G	3 (0.7%)
Cefalexin	2 (0.5%)
Pivmecillinam	1 (0.2%)
Cefotaxime	1 (0.2%)
Ceftriaxone	1 (0.2%)

*n* = number of cases; the cumulative total of all values within this table exceeds 100% as a consequence of the administration of multiple antibiotic agents in several patients.

**Table 5:** Baseline and follow-up culture results by microbial group in the 438 women with pPROM within our study cohort.

Culture Result			Follow-Up Microbial Analysis		*p*-Value
			No	Yes	
**Baseline microbial analysis**	*Lactobacillus* species	no	46 (20.4%)	1 (0.4%)	<0.001
		yes	170 (75.2%)	9 (4%)	
	Coagulase negative streptococci	no	77 (34.1%)	12 (5.3%)	<0.001
		yes	120 (53.1%)	17 (7.5%)	
	*Ureaplasma* species	no	124 (54.9%)	31 (13.7%)	0.289
		yes	41 (18.1%)	30 (13.3%)	
	*Mycoplasma hominis*	no	212 (93.8%)	4 (1.8%)	0.549
		yes	7 (3.1%)	3 (1.3%)	
	*Corynebacterium* species	no	164 (72.6%)	1 (0.4%)	<0.001
		yes	56 (24.8%)	5 (2.2%)	
	*Enterococcus* species	no	157 (69.5%)	6 (2.7%)	<0.001
		yes	59 (26.1%)	4 (1.8%)	
	Beta-hemolytic streptococci group B	no	209 (92.5%)	1 (0.4%)	<0.001
		yes	16 (7.1%)	0 (0%)	
	Other beta-hemolytic streptococci	no	216 (95.6%)	1 (0.4%)	0.021
		yes	9 (4%)	0 (0%)	
	*Viridans* streptococci	no	192 (85%)	8 (3.5%)	0.007
		yes	24 (10.6%)	2 (0.9%)	
	*Escherichia coli*	no	180 (79.6%)	17 (7.5%)	0.627
		yes	21 (9.3%)	8 (3.5%)	
	Other *enterobacterales*	no	201 (88.9%)	7 (3.1%)	0.134
		yes	15 (6.6%)	3 (1.3%)	
	*Gardnerella vaginalis*	no	170 (75.2%)	6 (2.7%)	<0.001
		yes	50 (22.1%)	0 (0%)	
	*Candida albicans*	no	199 (88.1%)	1 (0.4%)	<0.001
		yes	22 (9.7%)	4 (1.8%)	
	Non-albicans *Candida*	no	216 (95.6%)	0 (0%)	0.004
		yes	9 (4%)	1 (0.4%)	
	*Staphylococcus aureus*	no	219 (96.9%)	1 (0.4%)	0.219
		yes	5 (2.2%)	1 (0.4%)	
	Gram-positive anaerobes	no	198 (87.6%)	17 (7.5%)	0.169
		yes	9 (4%)	2 (0.9%)	
	Gram-negative anaerobes	no	132 (58.4%)	11 (4.9%)	<0.001
		yes	78 (34.5%)	5 (2.2%)	

**Table 6:** Detection rate of resistant pathogens in the baseline and follow-up vaginal cultures in the 438 women with pPROM within our study cohort.

Resistant Pathogens		Follow-Up Culture			*p*-Value
		No	Yes	Total	
**Baseline culture**	no	179 (79.2%)	27 (12%)	206 (91.2%)	0.024
	yes	12 (5.3%)	8 (3.5%)	20 (8.8%)	
	total	191 (84.5%)	35 (15.5%)	226 (100%)	

## Data Availability

Full data are available upon reasonable request to the corresponding author.

## Abbreviations

EONS	early onset neonatal sepsis
*E. coli*	*Escherichia coli*
pPROM	preterm premature rupture of membranes
spp.	species
